# Transitional B cells involved in autoimmunity and their impact on neuroimmunological diseases

**DOI:** 10.1186/s12967-020-02289-w

**Published:** 2020-03-17

**Authors:** Yang Zhou, Ying Zhang, Jinming Han, Mengge Yang, Jie Zhu, Tao Jin

**Affiliations:** 1 Department of Neurology and Neuroscience Center, The First Hospital of Jilin University, Xinmin Street 71#, Changchun, 130021 China; 2 Department of Neurobiology, Care Sciences and Society, Karolinska Institute, Stockholm, Sweden

**Keywords:** Transitional B cells, TrB-associated molecules, Autoimmune rheumatic diseases, Multiple sclerosis, Neuromyelitisoptica spectrum disorders

## Abstract

Transitional B cells (TrB cells) represent a crucial link between immature B cells in the bone marrow and mature peripheral B cells. Although TrB cells represent one of the regulatory B cell subpopulations in healthy individuals, the frequency of CD24^hi^CD38^hi^ TrB cells in circulation may be altered in individuals with autoimmune diseases, such as multiple sclerosis, neuromyelitisoptica spectrum disorders, systemic lupus erythematosus, Sjögren’s syndrome, rheumatoid arthritis, systemic sclerosis, and juvenile dermatomyositis. Although TrB cells play regulatory roles under inflammatory conditions, consequences of their functional impairment vary across autoimmune diseases. Since the origin, development, and function of TrB cells, especially in humans, remain unclear and controversial, this review aimed to discuss the characteristics of TrB cells at steady state and explore their role in various immune diseases, including autoimmune rheumatic diseases and neuroimmunological diseases.

## Introduction

B cells are centrally involved in the pathogenesis of autoimmunity, exerting diverse effects such as contributing to T cell activation through antibody production and antigen presentation. B cells also regulate immunological functions by suppressing T cell proliferation and producing pro-inflammatory cytokines, such as interferon-gamma (IFN-γ), tumor necrosis factor-α (TNF-α), and interleukin (IL)-17 [1, 2].

Transitional B cells (TrB cells) are bone marrow-derived, immature B cells, which are also considered to be precursors of mature B cells [3, 4]. In mice, approximately 10% of immature B cells overcome negative selection in the bone marrow and get transferred to the spleen as immature transitional B cells, where they eventually develop into mature B cells [4, 5]. However, in humans, the maturation of TrB cells may not be restricted to the spleen [5]. TrB cells account for approximately 4% of all CD19^+^ B lymphocytes in healthy individuals [6]. They are present in human bone marrow [5, 7], peripheral blood [8], cord blood [5, 6], and secondary lymphoid tissues (i.e., spleen [5, 9], tonsils [10], lymph nodes [5], and gut-associated lymphoid tissue [GALT]) [3], whereas mouse TrB cells are found only in the bone marrow, blood, and spleen, and not in the lymph nodes [4, 5]. CD24^hi^CD38^hi^ TrB cells are closely related to IL-10-producing regulatory B cells (Bregs) in terms of phenotypical and functional similarities [1]. TrB cells can also produce IL-10 and regulate CD4^+^ T cell proliferation and differentiation toward T helper (Th) effector cells [8]. The frequency of CD24^hi^CD38^hi^ TrB cells is elevated in those with systemic lupus erythematosus (SLE) [8, 11, 12], Sjögren’s syndrome (SS) [12], systemic sclerosis (SSc) [13], and juvenile dermatomyositis [14]. However, a low frequency of TrB cells has been noted in those with neuroimmunological diseases, including multiple sclerosis (MS) [15] and neuromyelitis optica spectrum disorders (NMOSDs) [16]. Accumulating evidence has suggested TrB cells to be part of the integrated immune system, in fact, these cells have been found to be involved in the pathogenesis caused by human immunodeficiency virus, type 1 [17] and hepatitis C virus infections [18], and *T. brucei* infections [19]. In addition, deletion of TrB cells resulted in a lack of mature B cell compartments during *T. brucei* infection, thus preventing the host’s ability of sustaining antibody responses against recurring parasitemic waves [19]. Since they are associated with several inflammatory diseases and are also found in circulation as well as tissues of healthy individuals, TrB cells are thought to perform distinct functions in immune-defense mechanisms.

Our understanding of TrB cells in healthy individuals as well as in those with diseases still remains incomplete. This may partially be due to their low frequency in circulation, in case of both mice and humans. In this review, we describe the origin, development, function, and associated molecules of TrB cells in the context of autoimmune diseases, with an emphasis on their neuroimmunological implications.

## The origin and development of transitional B cells

### Transitional B cells in mice

**Fig. 1:**
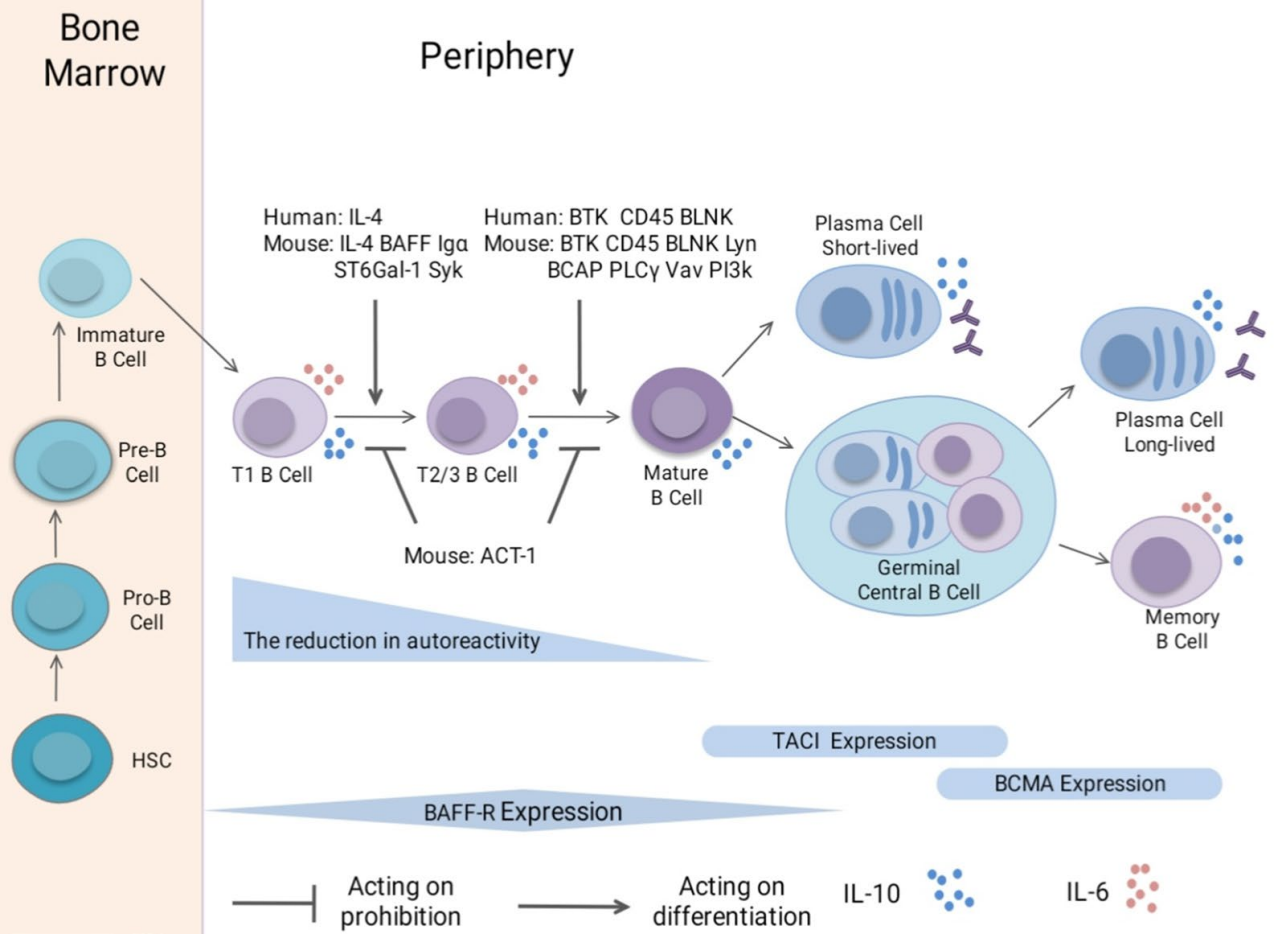
B cell differentiation pathways and expression of TrB-associated molecules. In the bone marrow (BM), HSCs undergo several stages of differentiation before they develop into immature B cells, including the pro-B and pre-B cell stages. The immature B cells emigrate from the BM, subsequently differentiating into T1 B cells in the periphery and then to the late TrB (T2/3 B) cells. This maturation step from T1 B cells to T2/3B cells requires IL-4, BAFF, Igα, ST6Gal-1, and Syk in mice and IL-4 in humans. The subsequent process of TrB cell differentiation into mature B cells requires BTK, CD45, and BLNK both in mice and humans, and Lyn, BCAP, PLCγ, Vav, and PI3K in mice. ACT-1 in mice plays a negative role in the development of TrB cells. Autoreactivity gradually reduces during B cell maturation, especially during TrB cell development. The late TrB cells develop into mature B cells, and give rise to either short-lived plasma cells or germinal center B cells. In the germinal center, they can undergo selection to differentiate into long-lived plasmablasts or memory B cells. B cells express three forms of BAFF receptors. BAFF-R is expressed on B cells from the TrB cell stage to the memory B cell stage in B cells, except in BM plasma cells, TACI is mainly expressed on memory B cells and some active mature B cells, whereas BCMA is expressed on memory B cells and plasma cells. IL-10 can be produced by TrB cells, mature B cells, plasmablasts, and memory B cells. IL-6 is expressed in TrB cells and memory B cells

Mouse B lymphocytes originate from hematopoietic stem cells (HSCs) in the bone marrow and fetal liver after birth, where they subsequently mature via immunoglobulin heavy chain and light chain recombination [20–22]. Based on cell surface phenotype and expression of B-lineage genes and of heavy chain and light chain, B cells in the bone marrow mainly include pre-B, pro-B, immature and recirculating B lymphocytes [23]. Of the 20 million IgM^+^ (B-cell receptor [BCR]^+^) B cells generated in the mouse bone marrow every day, approximately 10% enter circulation, and 1–3% reach the mature B cell pool [4]. The immature B cells transit to the marginal sinuses and red pulp of the spleen through the bone marrow sinusoids and bloodstream, after which the immature transitional 1 (T1) B cells migrate into the periarteriolar lymphoid sheath (PALS) of the white pulp in response to positive selection [24, 25]. BCR-mediated negative selection occurs at the T1 B cell stage, which serves to remove the self-reactive B cells; the remaining T1 B cells give rise to the late transitional B cells (T2/T3 B cells) [26, 27]. These gradually develop into naïve follicular mature (FM) or marginal zone (MZ) B cells and eventually into mature naïve B cells (Fig. 1) [28, 29].

Recent data have suggested TrB cell development to be modulated by various factors. First, it may be influenced by the integrity of BCR signaling [4, 9, 30, 31]. The BCR is composed of an Ig-α/Ig-β (CD79a/CD79b) heterodimer and a membrane-bound Ig molecule, which serves as a signal-transduction unit, linking the BCR with a complex of cytoplasmic signaling molecules [4, 9, 32]. For instance, mice lacking the cytoplasmic tail of Ig-α component of the BCR [4] or protein tyrosine kinase Syk [31] have shown restricted progression from T1 to T2 B cell subpopulations. CD45^−^ or Bruton’s tyrosine kinase (Btk)^−^ mutant CBA/N mice have shown arrested progression from T2 B cells into mature B cells in the spleen [9, 30]. Factors influencing the BCR signaling pathways may also limit various phases of TrB cell development [33–35]. For example, in signal-peptide-peptidase-like 2a (SPPL2a)^−/−^ mice, BCR signaling is impaired (to a great extent) by the accumulation of CD74 N-terminal fragment, which blocks B cell development at the T1 stage [33]. B cell development in B cell activating factor receptor (BAFF-R, BR3)^−/−^ mice was blocked progressing from T1 stage to T2 stage [34, 35]. The role of BAFF-R signaling in TrB cell generation is secondary to that of BCR signaling [36].

Second, negative and positive selection is important for the survival of both immature and mature B cells [37]. TrB cells, especially those in the T1 cell subset, are the targets of the negative selection in normal mice [23, 38]. Negative and positive selection of developing B cells promote a non-pathogenic B cell repertoire [21]. Negative selection occurs in BCR^+^ immature B cells through BCR-mediated apoptosis (central deletion), anergy, or receptor editing (secondary gene rearrangement), which removes self-reactive B cells [28, 39]. Although TrB cells are limited in terms of receptor editing, they maintain their capacity for negative and positive selection [39]. Data from some previous studies had suggested BCR signaling to regulate antigen-dependent positive selection of TrB cells [40, 41], thereby enhancing survival and/or clonal expansion [42, 43]. Kolhatkar et al. had demonstrated the positive selection of TrB cells expressing low-affinity self-reactive BCRs to be enhanced via the altered BCR and Toll-like receptor (TLR) signaling [44].

Finally, the splenic microenvironment is required for the B cell development; it affects the TrB cell compartment [9, 43]. Although mature B cells establish connections between lymphoid tissues and blood, T2 cells are restricted to the splenic follicles [9]. Thus, TrB cells develop into follicular or MZ B cells in different splenic compartments [45].

### Transitional B cells in humans

The process of B cell development in humans is similar to that in mice (Fig. 1); B cells originating from HSCs can be processed in the bone marrow, where they undergo heavy and light chain rearrangements before entering the peripheral compartment [1]. HSCs initially stem from common lymphoid progenitors and then develop into pro-B cells via rearrangement of the D and J segments following juxtaposition of a variable (V_H_) gene with the DJ_H_ element [46, 47]. Pro-B cells mature into pre-B cells via the rearrangement of μ–H-chain gene segments, which couple with a surrogate light chain [6, 46, 47]. After initial rearrangement of the light chain (V–L), human pre-B cells undergo 1 or 2 cell divisions and develop into immature B cells, which first express μ/κ or κ/λ surface IgM receptors [6, 10, 46, 47]. These immature T1 B cells are thought to emigrate from the bone marrow, subsequently differentiating into T2 B cells in the periphery, eventually giving rise to T3 B cells [48]. However, there is some disagreement regarding this process. TrB cells might be capable of departing from the bone marrow at either the T1 or T2 B cell stage, and some T1 B cells can differentiate into T2 B cells in the bone marrow, considering that similar proportions of T1 and T2 B cell subsets has been noted in normal human bone marrow [7]. In agreement with previous findings in mice, positive and negative selection may also regulate the development of human TrB cells [47]. In normal human peripheral blood, approximately 40% of TrB cells show autoreactive behavior; the frequency is reduced to 20% when they are differentiated into mature naïve B cells [47, 49]. TrB cell maturation requires Btk, demonstrating a possibility of BCR engagement, and is accompanied by improved survival [48]. BAFF promotes immature B cell selection and non-autoreactive immature B cells differentiation into TrB cells, although the role of BAFF in human TrB cell development has been controversial [36]. Some previous data had shown BAFF to have no effect on human TrB cells, although it serves as an efficient pro-survival factor for mouse TrB cells [5, 47, 48]. Some data has indicated that human T1 B cell proliferation to be clearly induced by BAFF, though to a lesser extent than that of human T2 B cells [50]. Therefore, during the process of TrB cell differentiation into mature naïve B cells, selection against self-reactive antibodies occurs and immune competence is gradually acquired in humans [49, 50].

Human TrB cells were first described in detail in 2005, and are often characterized by a CD24^hi^CD38^hi^ phenotype [1, 5]. Human CD24^hi^CD38^hi^ TrB cells represent approximately 4% of all CD19^+^ B cells in the peripheral blood, and constitute nearly 50% of B cells in cord blood and their proportion gradually declining during infancy [6]. The percentage of TrB cells in various normal mice tissues is different, and shown in Table 1 [4]. However, mouse TrB cells are not found in the lymph nodes owing to the absence of L-selectin expression in them [4]. In contrast, human TrB cells have been found in GALT, and previous data had indicated the T2 subset to be consistently enriched in GALT compared to the T1 subset [3]. Moreover, most T2 B cells isolated from human GALT were activated in response to intestinal bacteria, both in vitro and in vivo [3]. Therefore, the proportion of human CD24^hi^CD38^hi^ TrB cells may also differ within various tissues.

**Table 1:** Differences of phenotypes, locations, proportion of B cells, and subset ratios between mouse and human transitional B cells

	Mouse TrB cell	Human TrB cell
Phenotypes	T1: CD24^hi^CD21^lo^CD23^lo^IgM^hi^IgD^lo^ T2: CD24^hi^CD21^hi^CD23^hi^IgM^hi^IgD^hi^	T1: CD19^+^CD24^hi^CD21^lo^CD23^lo^IgM^hi^IgD^lo^ T2: CD19^+^CD24^hi^CD21^hi^CD23^hi^IgM^hi^IgD^hi^
	T1: CD24^+++^CD21^−^CD23^−^IgM^+++^IgD^−^CD93^+^CD62L^−^ T2: CD24^+++^CD21^++^CD23^++^IgM^+++^IgD^++^CD93^+^CD62L^−^ T3: D24^+++^CD21^++^CD23^++^IgM^++^IgD^++^CD93^+^CD62L^+^	T1: R123^+^CD38^+++^CD24^+++^CD10^+^IgD^+^CD27^−^ T2: R123^+^CD38^++^CD24^++^CD10^+^IgD^+^CD27^−^ T3: R123^+^CD38^+^CD24^+^CD10^+^IgD^+^CD27^−^
		T1: CD19^+^CD24^hi^CD38^hi^CD27IgM^hi^IgD^lo^CD10^hi^CD21^lo^CD32^hi^ T2: CD19^+^CD24^hi^CD38^hi^CD27IgM^in^IgD^in^CD10^in^CD21^lo^CD32^in^ T3: CD19^+^CD24^hi^CD38^hi^CD27IgM^lo^IgD^lo^CD10^lo^CD21^lo^CD32^lo^ CD27^+^TrB: CD19^+^CD24^hi^CD38^hi^CD27^+^
Locations	BM, PB, Spleen	BM, PB, CB and secondary lymphoid tissues (spleen, tonsil, lymph node and GALT)
The proportion of B cells	BM: 15–20%	BM: 6.3%
	PB: 15–20%	PB: 4%
	Spleen: 10–15%	CB: 50%
Subset ratios (TrB: FM B)	BM 1:0.6	BM 1:2.1
	PB 1:0.4	PB 1:2.2
	Spleen 1:3.7	Spleen 1:21.9

We along with other investigators had previously shown that Bregs contribute to the maintenance of immune tolerance and modulation of immune responses [1, 51–53]. Currently, human Bregs have been noted at different stages of B cell development and consist of various B cell subsets, namely CD24^hi^CD38^hi^ immature TrB cells [11], CD24^hi^CD38^lo^CD27^+^ memory B cells [54], and CD24^−^CD38^+^CD27^int^ IL-10-secreting plasmablasts [55]. Human IL-10-producing Bregs are enriched within the CD24^hi^CD38^hi^ immature TrB cell subset [1, 11]. These observations reveal a relationship between human TrB cells and Bregs. Taken together, a detailed understanding of TrB cell development and characterization would be beneficial for understanding mechanisms in autoimmunity and providing new ideas for potential treatment strategies.

## Phenotypes and functional properties of TrB cells

TrB cells can be separated into two or more distinct subsets, both in mice and humans (Table 1), based on their surface phenotypes and functional features [28]. Loder and colleagues had proposed two subtypes of mouse TrB cells, namely transitional 1 (T1) and transitional 2 (T2) B cells, depending on the expression status of CD21 and IgD [4]. Allman et al. had divided TrB cells into three subsets (T1 B cells, T2 B cells, and T3 B cells) based on the expression of AA4 (CD93), CD23, and sIgM [30]. Disagreement exists regarding the classification of human TrB cells. For instance, Sims et al. had separated them into T1 and T2 subpopulations, based on the expression level of IgD and CD38 and the minimal gradation of other surface markers such as CD24 [5]. Subsequently, human TrB cells were discriminated into three subsets, including T1 B cells, T2 B cells, and T3 B cells [50]. In 2016, Simon first proposed human CD24^hi^CD38^hi^ TrB cells to not contain only one subset, but rather 4 subsets based on CD27, IgM, IgD, CD10, CD21, and CD32 expression. T1-T3 B cells express low levels of CD27, whereas CD27^+^ TrB cells express CD27, CD24, and CD38 at high levels. In T1 B cells, the expression of IgM, CD10, and CD32 is high while that of IgD and CD21 is low. T2 B cells show moderate IgM, IgD, CD10, and CD32 expression and low CD21 expression. However, T3 B cells express IgM, IgD, CD10, CD21, and CD32 at low levels [8]. Notably, some studies indicated that CD27^+^ TrB cells serve as a type of Bregs and may not just being the transitional B cells [50, 56].

**Fig. 2:**
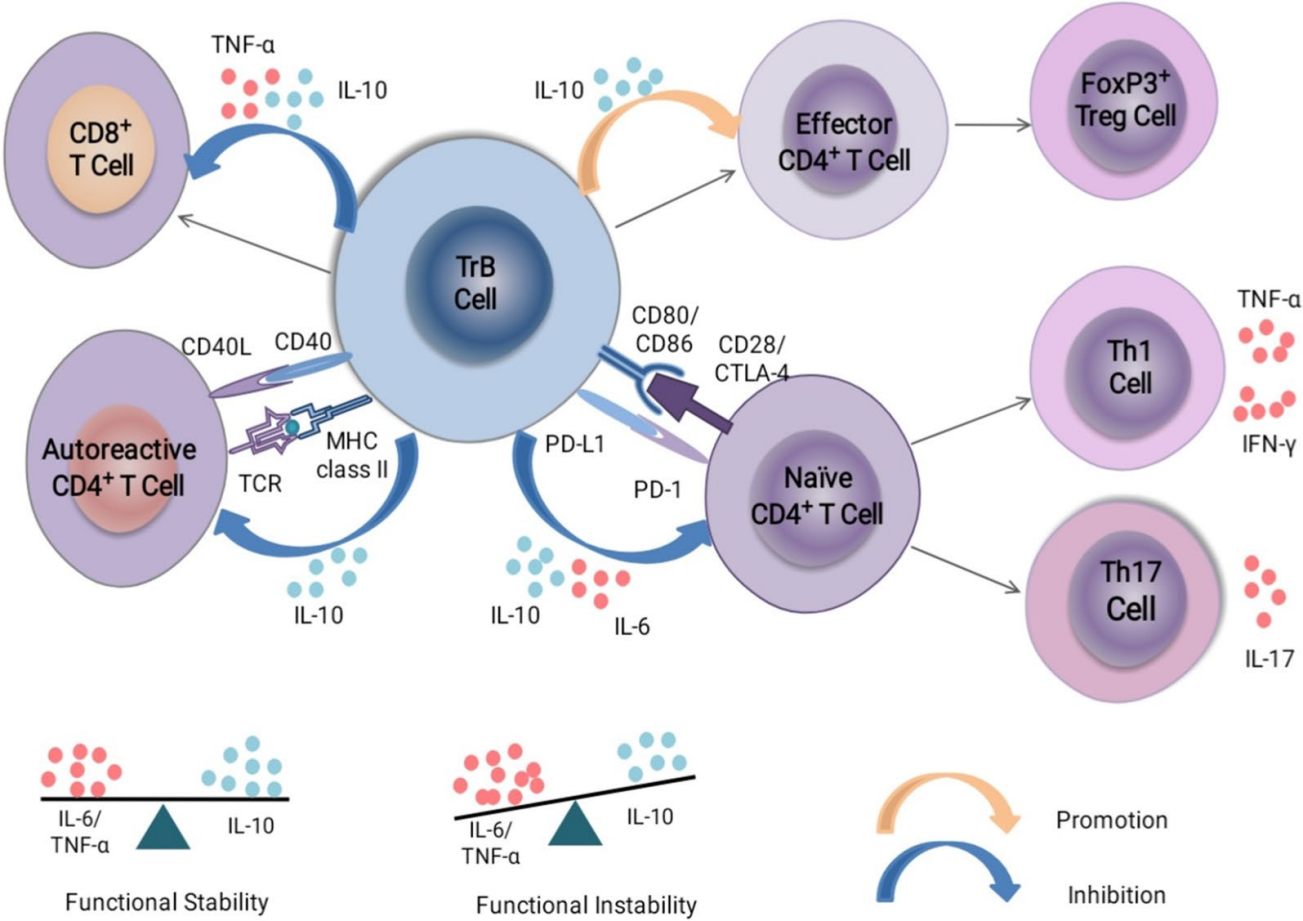
The main functions of transitional B cells. 1) TrB cells suppress the proliferation of autoreactive CD4^+^ T cells. 2) TrB cells prevent the differentiation of CD4^+^ T cells into Th1 and Th17 cells, thus limiting the production of excessive pro-inflammatory cytokines (TNF-α, IFN-γ, and IL-17). 3) TrB cells promote the conversion of effector CD4^+^ T cells into CD4^+^FoxP3^+^ Tregs. 4) TrB cells inhibit CD8^+^ T cell responses. The suppressive functions of TrB cells are partially dependent on the secretion of IL-10, which can down-regulate CD86 expression in an autocrine manner. TrB cells can also secrete pro-inflammatory cytokines, such as IL-6 and TNF-α. The imbalance between pro- and anti-inflammatory cytokine productions in TrB cells may affect their functional stability and thus participate in the development of immune diseases

In mice, T1 B cells clearly stop proliferating, and undergo apoptosis in response to BCR cross-linking, although discrepant findings have been reported for T2 B cells [28]. Some data have shown T2 B cells to proliferate and get induced to differentiate into follicular mature B cells in response to BCR engagement [4, 23], whereas other findings have suggested T2 B cells to not proliferate in response to the same signals [30, 57]. Mouse TrB cells are unable to proliferate following sIgM cross-linking in vitro [30]. However human TrB cells produce low amounts of Ig and exhibit less differentiation, proliferation, and chemotaxis in vitro than mature B cells [58]. Mouse splenic IL-10-producing Bregs include CD21^hi^CD23^hi^IgM^hi^ transitional 2-MZ precursor (T2-MZP) B cells [59] and CD1d^hi^CD5^+^ B cells [60]. T2-MZP B cells can suppress IFN-γ production by releasing IL-10 [59]. In addition, T2-MZP B cells also significantly reduce the proportion of CD4^+^IFN-γ^+^ and IL-17^+^ T cells, and contribute to differentiation into CD4^+^Foxp3^+^ T cells when cultured with effector CD4^+^CD25^−^ T cells [61, 62]. Similar to the observations in mice, human IL-10^+^ B cells with a transitional CD19^+^CD24^hi^CD38^hi^ phenotype have been found in peripheral blood of healthy individuals [11]. The main functions of human CD19^+^CD24^hi^CD38^hi^ TrB cells discovered at this time have been summarized in Fig. 2. First, IL-10 produced by TrB cells suppresses autoreactive CD4^+^ T cell proliferation [1, 11, 63]. Second, TrB cells suppress the production of pro-inflammatory cytokines by limiting the expansion of CD4^+^ Th1 cells (IFN-γ and TNF-α production) and CD4^+^Th17 cells (IL-17 production), which is dependent on IL-10, programmed cell death-ligand 1 (PD-L1), CD80, and CD86, but not on transforming growth factor-β (TGF-β) [1, 11, 56]. Third, TrB cells prevent the CD4^+^ T cells from differentiating into Th1 and Th17 cells and promote the conversion of effector CD4^+^ T cells into CD4^+^FoxP3^+^ Tregs while limiting the production of excessive pro-inflammatory cytokines [11, 64]. Finally, TrB cells inhibit CD8^+^ T cell responses and maintain invariant nature killer T (iNKT) cells [1, 65, 66]. Recent findings have revealed different subpopulations of TrB cells to have different functions. For example, T2/T3 B cells have a significant capacity to decrease CD4^+^ T cell proliferation (contrary to other subpopulations), and only CD27^+^ TrB cells significantly reduced TNF-α and IFN-γ production by CD4^+^ T cells [8]. In addition to producing anti-inflammatory factors, TrB cells can also secrete pro-inflammatory cytokines such as IL-6 and TNF-α [13, 14, 56, 67, 68]. Data from previous studies had shown the frequency of IL-6-producing TrB cells to be elevated in SLE, SS, and other immune diseases [13, 14, 68]. IL-6 restricts the differentiation of CD4^+^ T cells into Tregs and induces autoreactive Th1/Th17 T-cell responses [69, 70]. Therefore, the pro- and anti-inflammatory cytokines produced in TrB cells may affect their functional stability [13, 67].

In summary, TrB cells comprise different subsets, showing unique phenotypic and regulatory functional profiles. The frequencies and functions of TrB cells may be altered in different autoimmune diseases.

## The development and function of TrB-associated molecules

### BAFF: B cell activating factor

BAFF (also known as BLys, TNFSF13B, TALL1, and ZTNF4) is a member of the TNF family that can be induced by various inflammatory cytokines, particularly IFN-γ [71–73]. It is mainly produced by some lymphocytes (activated B cells and T cells), innate immune cells (neutrophils, macrophages, monocytes, dendritic cells, and follicular dendritic cells), and non-lymphoid cells (astrocytes, synoviocytes, epithelial cells, and cytotrophoblasts) [71]. BAFF promotes B cell maturation and regulates immune responses via three receptors: transmembrane activator and CAML interactor (TACI, also known as TNFRSF13B and CD267), BAFF receptor (BAFF-R, also known as BR3, TNFRSF13C, and CD268) and B cell maturation antigen (BCMA, also known as TNFRSF17 and CD269) [71, 74]. In humans, TACI is mainly expressed in memory B cells and in some active mature B cells, whereas BCMA is presented in memory B cells and tonsillar, splenic, and bone marrow plasma cells (Fig. 1) [71, 74]. BAFF-R is expressed from TrB cell stage to memory B cell stage, in absence of bone marrow plasma cells, where it is expressed at higher levels in T2 B cells than in T1 B cells or mature B cells (Fig. 1) [71, 74].

Recently, many studies have been shown the role of BAFF in mice TrB cell development. Such as, gene-knockout studies have demonstrated the differentiation of T1 B cells into T2 B cells in BAFF^−/−^ or BAFF-R^−/−^ mice to be mostly blocked, resulting in fewer T2/3 and mature B cells [34, 46, 74]. Recent data have also suggested BAFF to promote the differentiation of T1 B cells into T2 B cells, and BAFF-R to be necessary for survival during the transition from T2 B cells to MZ B cells in mice [34, 75]. Clearly, BAFF binding to BAFF-R promotes the survival of T2, T3, and mature B cells [34, 75]. In vitro and in vivo data have shown BAFF and tonic BCR signaling to cooperate to contribute the differentiation of CD23^−^ TrB into CD23^+^ TrB cells [36]. Additionally, a synergy between optimal BAFF-R signaling and tonic BCR signals can contribute to positive selection and differentiation of non-autoreactive immature B cells into TrB cells [36]. BAFF-R expression levels are regulated by BCR signaling in TrB cell subtypes [37]. But, some observations in humans have been shown differences or inconsistencies compared with mice studies. Furthermore, the role of BAFF in human TrB cell development has been controversial, but BAFF promotes immature B cell selection and non-autoreactive immature B cells differentiation into TrB cells in humans [36]. Some previous data had shown BAFF to have no effect on human TrB cells [5, 47, 48], while some data has indicated that human T1 B cell proliferation to be clearly induced by BAFF, though to a lesser extent than that of human T2 B cells [50]. BAFF, which is necessary for B cell survival and maturation, suppressed IFN-γ production in vitro and inhibited the proliferation of anti-CD3-stimulated CD4^+^CD25^−^ T cells by inducing Bregs [2]. BAFF overexpression in serum promoted the malignant proliferation of B cells, leading to autoimmune diseases [34].

### IL-10

IL-10 is a broad-spectrum immunomodulatory cytokine that modulates antigen presentation of cells, inhibits T cell proliferation, and maintains Treg functions [76]. IL-10 is produced by most types of hematopoietic cells in mice, and almost all types of B cells in humans [2, 8]. IL-10-producing B cells were shown to be available in the TrB cell compartment [1]. In other words, TrB cells seem to exert their immunosuppressive effects mainly by producing IL-10. For example, IL-10 produced by CD19^+^CD24^hi^CD38^hi^ TrB cells down-regulate CD86 in an autocrine manner, eventually suppressing CD4^+^ T cells proliferation and reducing IFN-γ and TNF-α production [63, 64]. In addition, human TrB cells produce more IL-10 after CD40 engagement, demonstrating IL-10 as an essential factor for various co-stimulatory molecules [64, 76]. Taken together, the findings indicate IL-10 to exert important immune regulatory functions in TrB cells.

### IL-4

IL-4 is a multifunctional cytokine that promotes mature B cell activation and induces B cell differentiation, proliferation, and antibody secretion by acting as a cofactor for lipopolysaccharides, CD40L, and antigen stimulation [77, 78]. IL-4 stimulates the development of CD23^−^ TrB cells into CD23^+^ TrB cells, an effect comparable to that driven by BAFF [77]. However, IL-4 was found to render bone marrow TrB cells refractory to anti-IgM apoptotic signals, which was distinct from BAFF [77]. Thus, IL-4 and stromal cells play major roles in improving the survival of human T1 B cells and promoting their maturation [5].Taken together, the findings revealed IL-4 to contribute to TrB cell differentiation and maturation both in mice and humans.

### IL-6

IL-6 is a multi-functional cytokine involved in the pathogenesis of autoimmune diseases [79]. It is produced by various types of immune and stromal cells [79, 80]. Some studies have shown that IL-6 may promote the differentiation of naive CD4^+^ T cells into Th17 cells in both mice and humans [81, 82]. IL-6 blockade was used in experimental autoimmune encephalomyelitis (EAE), an animal model of MS. It was found that it can suppress Th1 and Th17 cellular responses, leading to reduced T cells infiltration into the central nervous system (CNS) during EAE period [83]. IL-6 is also produced by TrB cell and the frequency of IL-6-producing TrB cells was seen to be elevated in patients with SLE, SS and other immune diseases [13, 14, 68].

### CD40

CD40 is a co-stimulatory member of the tumor necrosis factor receptor (TNFR) super family and is expressed in both immune and non-immune cells [84]. CD40L (CD154) is the major ligand of CD40, and is predominantly expressed in activated CD4^+^ T cells; CD40-CD40L interactions can inhibit T cell proliferation and differentiation into Th1 cells [84, 85]. Some studies have shown that human TrB cells produce more IL-10 and express higher levels of the IL-10 receptor after CD40 engagement when compared with other B cell subsets, leading to a decreased expression of CD86 in TrB cells and proliferation of CD4^+^ T cells [11, 63, 85]. However, CD40L-blocking antibodies were used when co-cultured healthy TrB cells with CD4^+^CD25^−^ effector T cells, which were unable to affect the production of IFN-γ or TNF-α [11, 85]. In a mouse model of EAE, B cells from CD40^−/−^ mice failed to produce IL-10 or induce disease remission [86]. In summary, CD40-CD40L signaling plays a critical role in maintaining appropriate immune responses in both mice and human.

### ACT-1

ACT-1 (Traf3ip, Ciks) contains a helix-loop-helix motif at its N terminus and a coiled-coil at the C terminus, and it plays a negative role in autoimmunity through its differential impacts on both CD40-CD40L and BAFF-mediated pathways [87, 88]. CD40 and BAFF signaling appear to be necessary for B cell survival and maturation, especially in the transitional stages [34, 86]. In ACT-1 deficient mice, the number of T1 and T2 B cells in the spleen is significantly expanded; BAFF stimulation induces more T1 cell survival and efficiently promotes T1 cell maturation into T2 cell in vitro [88, 89]. During transitional stages, ACT-1 potentially promotes the elimination of autoreactive B cells and regulation of peripheral B cell homeostasis [89]. Together, the findings support the view that the adaptor molecule ACT-1 is a key regulator of TrB cells.

### ST6GaL-1: ST6Beta-galactosamide alpha-2,3-sialyltransferase 1

ST6GaL-1 is a sialyltransferase, and its cognate α-2,6-sialyl catalytic product can interact with CD22 (B cell complex accessory molecule) [90]. ST6Gal-1 is widely distributed in many tissues with high levels being noted in B cells, liver, and lactating mammary glands [91, 92]. Extrinsic sialylation by ST6GaL-1 contributes to the development of TrB cells, BAFF and BCR-mediated signaling [93]. Co-culturing immature B cells with ST6Gal-1 resulted in an increased number of TrB cells, demonstrating circulating ST6GaL-1 as a pro-survival factor important for BAFF utilization by non-autoreactive TrB cells [93]. Some studies have shown CD22 to recognize the a2,6-sialic acid produced by ST6Gal-1, indicating that the humoral immunodeficiency may exist in ST6GaL-1^−/−^ animals [94, 95].

## Transitional B cells in autoimmune rheumatic diseases and neuroimmune disorders

### TrB cells and TrB-associated molecules in autoimmune rheumatic diseases

At present, most studies on TrB cells and TrB-associated molecules in non-neuroimmune disorders are related to autoimmune rheumatic diseases (AIRDs); however, the etiology of most AIRDs still remains poorly understood. AIRDs are known to produce pathogenic antibodies and cause damage to different target organs and tissues [64, 68, 84, 96, 97]. For example, SLE can affect most organs in humans, whereas SS mainly affects the exocrine glands (labial glands and lacrimal glands) and RA primarily involves the joints and surrounding tissues [64, 68, 84]. Related observations have revealed that B cell-targeting therapy can control the development of such diseases [68, 98]. Therefore, B cells play important roles in the pathogenesis of AIRDs. The abundance of TrB cells and their impairment of immune-regulatory functions may vary in different diseases. The frequency of TrB cells is expanded in SLE [8, 11, 12], SS [12], and SSc [13], whereas it is decreased in RA before treatment [12, 64].

Like Bregs, TrB cells exert a protective role, both in healthy individuals and in those with AIRDs, partially via IL-10 production. However, isolated TrB cells in patients with SLE fail to inhibit IFN-γ and TNF-α production by CD4^+^ T cells [11], while TrB cells in healthy individuals can maintain the balance between Th1/Th17 and Tregs populations [64]. Furthermore, TrB cells from active RA cases restrict CD4^+^ T cell differentiation into Tregs and Th17, while maintaining the ability to inhibit Th1 development [64]. Besides IL-10, other TrB cell-associated molecules also play roles in auto-immune diseases. More IL-6-producing TrB cells are found in those with SLE and SSc than in healthy controls, which are positively correlated with disease activity and serum anti-dsDNA autoantibody titers in SLE cases [13, 68]. IL-6 is a pro-inflammatory cytokine that suppresses the generation of Tregs and contributes to the production of CD4^+^ Th17 cells [69]. Therefore, TrB cells can produce both anti-inflammatory and pro-inflammatory cytokines to exert their immunomodulatory functions. In some immune diseases, the frequency of TrB cells is augmented and positively correlated with the disease activity, although their immune-regulatory function is impaired [11, 13]. Mounting evidence has suggested interferon (IFN) signature, TLR9 response impairment, and CD19 down-regulation of TrB cells are involved in those with SLE, thereby interfering with the removal of autoreactive B cells at the TrB cell stage, and leading to the production of pathogenic antibodies by the surviving autoreactive B cells [68, 96]. In addition, SLE plasma with IFN-α efficiently induces IL-6 expression in TrB cells, indicating IFN-α to possibly play an important role in stimulating TrB cells to produce IL-6 [68].

Taken together, these findings indicate that the participation of TrB cells in pathogenesis of AIRDs, and reveal that impairment of their immune-regulatory functions may reflect their inability to prevent the processes underlying autoreactive responses and inflammation.

### TrB cells and TrB-associated molecules in MS and EAE

MS is considered a chronic inflammatory demyelinating disease of the CNS, leading to the loss of myelin, along with axonal and neuronal degeneration [51, 99–102]. The pathologic features of MS are typically characterized by the dissemination of time and space [101, 102]. Historically, the factors considered to induce MS include the activation of pathogenic Th1, Th17, and CD8^+^ myelin auto-reactive T cells, in addition to genetics, and environmental factors, and infections [99, 100, 103]. In support of this animal model, EAE also can be induced by myelin-specific CD4^+^ T cells and CD8^+^ T cells [99]. Recently, therapies targeting B cells and B cell cytokines (rituximab, ocrelizumab, ofatumumab, and tabalumab) have been shown to suppress MS disease activity [101, 102]. Furthermore, B cell depletion in MS can reduce auto-antibodies and pro-inflammatory cytokines produced by B cells and impair antigen presentation [103, 104]. B cell infiltration was found in the brain tissues of patients with MS, and oligoclonal bands (clonal IgG) and antibody-secreting plasma cells have been discovered in the cerebrospinal fluid [99, 105]. Meningeal ectopic B cell follicles were formatted in secondary-progressive MS [101]. Collectively, these observations demonstrated the participation of B cells in the pathogenesis of MS. Recent research has shown B cells to be virtually depleted after anti-CD20 treatment in EAE, then after 6–8 weeks, they gradually reappeared in the bone marrow, spleen, lymph nodes, and blood [106]. Surprisingly, splenic TrB and follicular B cells were found to be almost absent in EAE treated with anti-CD20, at 8 weeks post-treatment [106].

In addition to their involvement in MS pathogenesis, B cells also exert immune-regulatory effects by producing anti-inflammatory cytokines directly or indirectly [102, 107]. Bregs play a regulatory role in MS by helping with the maintenance of immune tolerance [1, 108]. In mice, IL-10-producing CD1d^hi^CD5^+^ B cells play a more substantial role in suppressing EAE initiation and development than other B cells [60, 109]. TrB cells account for a considerable proportion of functional Breg cells [1, 11]. Significantly fewer TrB cells were detected in those with MS/clinically isolated syndrome (CIS) than in healthy individuals, and the frequency of TrB cells was increased after related treatments (e.g., thymosin-α1, fingolimod) [15, 110, 111]. Similar to that of TrB cells, the frequency of other circulating B-cell subsets (naïve, MZ-like, and memory B cells) in peripheral blood of MS/CIS cases were comparable to those in healthy controls [15]. Some data further demonstrate TrB cells to be capable of homing to inflamed sites in the CNS through up-regulated expression of α4 and β1 integrins during the early phase of MS [15]. TrB cells in the peripheral blood of those with MS who were treated with fingolimod, produced more IL-10 and less TNF-α than memory and mature naïve B cells, and expressed low levels of CD80 [111]. Related studies have shown the immune-regulatory role of Bregs to be partly mediated through the production of IL-10, IL-35, TGF-β and PD-L1 [63, 64, 103]. In EAE, CD40 signaling is required for inducing IL-10 competence [86, 112]. Subsequently, IL-21 drives the expansion of IL-10-producing B cell as well as generation of effector cells [86, 112]. IL-6-producing B cells are involved in the pathogenesis of EAE/MS [70, 113]. Over 65% of IL-6 is primarily produced by B cells, which exacerbates EAE, augments the autoreactive T cell responses of Th1 and Th17 types, and inhibits the generation of Tregs [98, 114].

TrB cells, like Bregs, play an immune-regulatory role in MS/EAE; however, further research would be required to show how TrB cells activate, proliferate, and function in MS. Many approved disease-modifying drugs for MS have been able to reduce the number of relapses and decrease activity of the disease; however, further research can focus on the understanding of pathogenic mechanisms underlying MS/EAE in order to explore the development of more specific and effective therapeutic approaches.

### TrB cells and TrB-associated molecules in neuromyelitis optica spectrum disorders

NMOSDs are idiopathic inflammatory demyelinating diseases affecting the CNS, and are clinically characterized by longitudinally extensive transverse myelitis (LETM) and severe optic neuritis (ON), traditionally considered a variant of Asian MS [115–118]. Lennon et al. first discovered a serological marker of neuromyelitis optica (NMO), known as the aquaporin-4 antibody (AQP4-Ab) or NMO-IgG, making NMO independent of MS [119]. The identification of AQP4-Ab broadened the spectrum of NMO [116, 120]. AQP4-Ab is involved in the pathogenesis of NMOSDs, depending on the presence of B cells, T cells, and complement, suggesting NMOSDs to possibly be primarily mediated by humoral immunity [117, 121]. NMOSDs cases can be associated with other autoimmune diseases such as SLE, SS, and thyroiditis, implying that NMOSDs may involve a mechanism resembling that of autoimmune diseases [115, 117]. Like SLE and other autoantibody-mediated immune diseases, NMOSDs cases have deficiencies in the integrity of the central and peripheral B cell tolerance checkpoints [122]. In addition, impaired peripheral B cell tolerance checkpoints interfere with the removal of autoreactive B cells, leading to increase the reservoir self-reactive new emigrant/transitional and mature naive B cells, and this process has relation with the production of pathogenic anti-AQP4 autoantibody [122].

Currently, only a few studies have been conducted to explore the roles of TrB cells and TrB-associated molecules in NMOSDs. Specifically, fewer CD19^+^CD24^hi^CD38^hi^ TrB cells were found in those with NMOSDs than in those with MS [16, 52]. The frequencies of CD19^+^CD27^+^ memory B cells and mature B cells are not significantly different between those with NMOSDs and HC, whereas the percentage of CD19^+^CD5^+^CD1d^hi^ Bregs decreased in NMOSDs cases [16, 52]. The proportion of TrB cells has been demonstrated to be lower in AQP4-positive group than in AQP4-negative group [16, 52]. Moreover, the frequencies of IL-10-expressing B cells among all lymphocytes are reported to be lower in those with NMOSDs than in those with MS and in healthy individuals [16]. This indirectly shows the impairment of immunomodulatory function of TrB cells in NMOSDs due to a reduction of TrB cells and/or IL-10; IL-10 production in TrB cells was not measured in most studies. Rituximab treatment in those with NMOSDs lowered the number of CD27^+^ memory B cells and increased the number of CD24^hi^CD38^hi^ TrB cells [123]. Related studies also showed elevation of BAFF, IL-6, and IL-21, both in serum and CSF of NMOSDs cases, although expression of these molecules in TrB cells still remains unclear [117, 121, 124]. AQP4-Ab is mainly derived from a subtype of B cells, CD27^hi^CD38^hi^CD180^−^ B cells, survival of which depends on signaling via the IL-6 receptor, thus indicating IL-6-dependent B cells to possibly be associated with NMOSDs pathogenesis [117]. Korn and colleagues had provided evidence of IL-6 and IL-21 suppressing Tregs production, despite inducing IL-17 secretion from CD4^+^ Th17 cells [69]. Further studies would be required to explore the mechanism driving the IL-6 production of TrB cells in those with NMOSDs. If the proportion of IL-6-producing TrB cells is elevated in NMOSDs cases, then IL-6 secretion may impair the immune-regulatory function of TrB cells.

In summary, TrB cells and TrB-associated molecules are involved in the pathogenesis of NMOSDs. However, further research would be required for determining their immunomodulatory roles in NMOSDs cases and for clarifying whether they are involved in AQP4-Ab production. These considerations are important for understanding whether TrB cells and TrB-associated molecules can serves as new therapeutic targets for those with NMOSDs.

## Conclusion and perspective

Although recent studies have demonstrated regulatory roles of TrB cells, the same may vary across different autoimmune diseases due to the production of diverse levels of anti-/pro-inflammatory cytokines, or induction of B cell immune-tolerance defects, leading to increasing autoreactive B cells and pathogenic antibodies. Further studies focusing on the activation, proliferation, and exact functional mechanisms of TrB cells in healthy individuals and in those with various immune/inflammatory diseases would be necessary in the future. Success in such endeavors may help in clarifying the role of TrB cells as disease biomarkers and/or therapeutic targets.

## Data Availability

Not applicable.

## Abbreviations

TrB cells: Transitional B cells
IFN-γ: Interferon-gamma
TNF-α: Tumor necrosis factor-α
IL: Interleukin
GALT: Gut-associated lymphoid tissue
Bregs: Regulatory B cells
Th: T helper
SLE: Systemic lupus erythematosus
SS: Sjögren’s syndrome
SSc: Systemic sclerosis
MS: Multiple sclerosis
NMOSDs: Neuromyelitis optica spectrum disorders
HSCs: Hematopoietic stem cells
BCR: B-cell receptor
T1: Transitional 1
T2: Transitional 2
T3: Transitional 3
PALS: Periarteriolar lymphoid sheath
FM: Follicular mature
MZ: Marginal zone
Btk: Bruton’s tyrosine kinase
SPPL2a: Signal-peptide-peptidase-like 2a
BAFF-R, BR3: B cell activating factor receptor
TLR: Toll-like receptor
T2-MZP: Transitional 2-MZ precursor
PD-L1: Programmed cell death-ligand 1
TGF-β: Transforming growth factor-β
iNKT: Invariant nature killer T
TACI: Transmembrane activator and CAML interactor
BCMA: B cell maturation antigen
NIK: NF-κB-inducing kinase
EAE: Experimental autoimmune encephalomyelitis
CNS: Central nervous system
TNFR: Tumor necrosis factor receptor
ST6GaL-1: ST6Beta-galactosamide alpha-2,3-sialyltransferase 1
AIRDs: Autoimmune rheumatic diseases
IFN: Interferon
Clonal IgG: Oligoclonal bands
CIS: Clinically isolated syndrome
LETM: Longitudinally extensive transverse myelitis
ON: Optic neuritis
AQP4-Ab: Aquaporin-4 antibody
BM: Bone marrow
PB: Peripheral blood
CB: Cord blood
